# Rapid and economic fabrication approach of dielectric reflectors for energy harvesting applications

**DOI:** 10.1038/s41598-020-73052-w

**Published:** 2020-09-28

**Authors:** Venkatesh Yepuri, R. S. Dubey, Brijesh Kumar

**Affiliations:** 1 Department of Nanotechnology, Swarnandhra College of Engineering and Technology, Seetharampuram, Narsapur, A.P. India; 2 Amity Institute of Nanotechnology, Amity University, Gurgaon, Haryana India

**Keywords:** Energy harvesting, Metamaterials

## Abstract

Dielectric reflectors are the passive components that have their potential demands for various purposes, such as back-end reflector in solar cells, the band pass filters in optical instruments, thermal reflector and so on. Though well-established techniques for manufacturing such reflectors are available, the demand for their low-cost production with a minimum number of coatings has attracted the attention of the scientific community. In this framework, this paper addresses the process optimization for the low-cost and rapid fabrication of dielectric TiO_2_/SiO_2_ reflectors with 100% reflectance. Numerous studies are carried out to explore the structural, morphological, and optical characteristics of reflectors. We summarize that the desired reflection band of a selective-wavelength range can be realized by varying the precursor and catalyst concentrations, annealing cycle, and the spin rate. With this, we noticed the shifting of reflection window from the visible (Vis) to near-infrared (NIR) wavelength region using reflectors of merely 2.5 stacks of TiO_2_/SiO_2_ films. We also performed the thermal response of the reflector by radiating an infrared light source and observed an exceptional performance indicating its thermal shielding application.

## Introduction

Dielectric reflectors or mirrors are the passive optical/photonic components that have been demanded in optical and photonic integrated circuits. These mirrors have also been demanded their potential applications in many optical measuring instruments like spectrophotometer, Raman spectrometer etc. Over the metallic reflectors, these are lightweight and lossless, depending upon the followed fabrication technique. The dielectric reflectors/multilayer structures based on alternate films of TiO_2_/SiO_2_, ZrO_2_/ZnO, SnO_2_/SiO_2_ etc. have been investigated for their optical, structural, and morphological studies^1–3^. To fabricate such reflectors, various techniques such as E-beam evaporation, molecular beam epitaxy, chemical vapor deposition, dip coating, RF sputtering, and sol–gel spin coating are being employed and reported in the literature^4–8^. In spite all, the sol–gel spin coating technique is the easiest and in-expensive one.

For the multilayer dielectric reflectors, the alternate films of TiO_2_/SiO_2_ are the suitable choice due to their significant refractive index contrast, which governs the optical property. Choi et al. reported the increased optical reflectance in the near-infrared wavelength region using TiO_2_/SiO_2_ optical filters prepared by the sol–gel and spin coating processes. It is a well-known phenomenon that the porosity of the film can be altered via refractive index variation and accordingly, the distinct light behavior can be attained. On this fact, fabrication of SiO_2_ film using base-and acid-catalysts was explored while base-catalyst based SiO_2_ film showed the small refractive index value. With this optimized process, reflector based on (TiO_2_/SiO_2_)_1.5_ stacks showed 62.87% reflectance in the near-infrared region as compared to the same structure prepared with SiO_2_ film based on acid-catalyst. Besides, this approach was useful to overcome the demand for more number of stacks^9^. Anaya et al. explored a similar approach for the variation of the refractive index of SiO_2_ film. To attain this, an alternate preparation of porous-SiO_2_ and dense-TiO_2_ films was preferred. The DBR based on (TiO_2_/SiO_2_)_3.5_ stacks were fabricated and endorsed as much as 90% reflectance in the visible wavelength range^10^. Delamination of either the film is a severe problem during the preparation of TiO_2_/SiO_2_ multilayer structure as each film undergoes sintering at a particular temperature for the number of times. In this contrast, Han et al. addressed the issue of delamination by exploring the hydrophobicity of SiO_2_ film. After optimizing the sintering process, they could attain the hydrophilic property of SiO_2_ film and later successfully fabricated the multilayer TiO_2_/SiO_2_ structure^11^. Dubey et al. prepared and studied the TiO_2_/SiO_2_ multilayer structures by varying the number of stacks. The reflectance was found to be increased as a function of a number of stacks, and maximum 90% was obtained from the 7-stacks based reflector. Further, they presented the modeling of silicon solar cells using (TiO_2_/SiO_2_)_7_ stacks and endorsed the improved light absorption in the visible spectral range^12^. Similarly, TiO_2_/SiO_2_ multilayer structure was prepared to improve the light absorption in the dye-sensitized solar cell. Lee et al. fabricated the reflector based on (TiO_2_/SiO_2_)_4_ stacks on stainless steel substrate by RF magnetron sputtering process at substrate temperature 600 °C. The prepared structure showed the reflection band in the spectral range from 400–550 nm, which is promising for the dye-sensitized solar cells as dye absorption is dominant in this wavelength region^13^^.^ In dye-sensitized solar cells (DSSCs), the light scattering mechanism is significant to increase the light-harvesting. Yasuda et al. prepared the TiO_2_/SiO_2_ multilayer flakes having its 100% reflectance in the visible wavelength range and employed in the DSSCs, which showed the enhanced short-circuit current^14^. An omnidirectional reflector is nothing but the reflector which has 100% reflectivity in a spectral range regardless of the incident angle of light. Jena et al. fabricated the omnidirectional reflector with the alternate deposition of TiO_2_ and SiO_2_ layers by pulsed DC magnetron and RF magnetron sputtering, respectively. The experimental result was found consistent with the simulated one with its omnidirectional photonic band from wavelength range 592–668 nm for the incident angle from 0° to 70°^15^. Nagayoshi reported the preparation of a scattering back reflector of TiO_2_-nanoparticles/SiO_2_ film by chemical routes. Remarkably, the prepared TiO_2_/SiO_2_ multilayer structure showed 90% reflectance in the infrared wavelength range^16^. Despite the sol–gel spin coating process, the dip-coating technique is also employed for the fabrication of TiO_2_/SiO_2_ Bragg reflectors. Sedrati et al. studied the multilayer structure of TiO_2_/SiO_2_ by varying the number of stacks. Regardless of the number of stacks, the XRD analysis exhibited the anatase and rutile-TiO_2_ phases, while the Si–O–Ti vibration peak evidenced by the FTIR investigation. Typically, the transmittance was found to be decreased with the increased number of stacks^17^. Using the dip-coating method, Yuehui et al. fabricated and characterized the TiO_2_/SiO_2_ multilayer structures of seven stacks prepared on the silicon substrate. By designing approach, they tuned the quarter wavelength thickness and fabricated the reflector having 65% reflectance from blue-green to infrared region at their center wavelengths 800 and 1600 nm, respectively^18^.

In this article, we present the process optimization for the low-cost and rapid fabrication of dielectric multilayer reflectors with the minimum number of TiO_2_/SiO_2_ stacks/bilayers. To the best of our knowledge, no such systematic process optimization study has been reported for the fast and cost-effective fabrication of Vis–NIR reflectors. In “Experimental details” section presents the details of chemicals and experimental processes for the preparation of dielectric reflectors. In “Results and discussion” section describes the obtained results. Lastly, “Conclusions” section concludes the study.

## Experimental details

### Chemicals

Titanium isopropoxide (TTIP, Sigma-Aldrich) and tetraethyl orthosilicate (TEOS, Sigma-Aldrich) were used as the source of Ti and Si, respectively. The ethanol (EtOH, Changshu Hongsheng Fine Chemicals) was used as the solvent, while hydrochloric acid (HCL, Fischer Scientific) and acetic acid (AcAc, Sisco Research Laboratories) were used as the catalysts. All chemicals were of analytical grade and used as procured.

### Sol–gel and spin coating processes

For the sol–gel synthesis, the following two parameters were studied, and the processes are discussed below.

- *Precursor concentration* For the preparation of TiO_2_ and SiO_2_ solutions, two different concentrations of the respective precursors were taken. TiO_2_ solutions were prepared by mixing EtOH:HCL:TTIP in the molar ratios 20:0.3:1 and 20:0.3:1.5. In the same way, EtOH:HCL:TEOS was preferred in the molar ratios 20:0.3:1 and 20:0.3:1.5 for the preparation of SiO_2_ solutions.
- *Catalyst concentration* The catalyst concentration was varied by mixing AcAc and HCL with predefined volumes. To prepare the TiO_2_ solution, EtOH:AcAc:HCL:TTIP were preferred in the molar ratio 10:1.2:0.1:1.2 while for the preparation of SiO_2_ solution, the preferred molar ratio of EtOH:AcAc:HCL:TEOS were 10:1.2:0.1:1.2.

After preparing the above solutions, various periodic multilayer structures of TiO_2_ and SiO_2_ films were deposited on the cleaned glass substrates by the spin coating process.

Further, the following two parameters were considered to study their effect on the structural, morphological, and optical properties of TiO_2_/SiO_2_ dielectric reflectors.

- *Heat treatment process* The annealing of the film was carried out in two ways, (1) annealing after depositing the individual film of TiO_2_ and SiO_2_ at temperature 500 °C for 1 h and, (2) annealing at temperature 500 °C for 1 h followed by pre-baking each film of TiO_2_ and SiO_2_ at temperature 100 °C for 30 min.
- *Spin rate/speed* To study the influence of thickness variation, the spin rate was varied to 2000 RPM, 3000 RPM, and 4000 RPM.

After preparation of TiO_2_/SiO_2_ reflectors, various investigations were carried out using X-ray diffraction (XRD, Bruker AXS D8 Advance, Germany), Fourier-transform infrared spectroscopy (FTIR, Bruker vertex 70, Germany), Ultraviolet–visible spectroscopy (UV 1800, Shimadzu, Japan) and field-emission scanning electron microscopy (FESEM, ZIESS, Germany). Hereafter, various prepared samples were named as: Low-P/High-P: low/high precursor concentration, HCL/HCL+AcAc: HCL/HCL+AcAc based catalyst, annealed/baked+annealed: only annealed at temperature 500 °C for 1 h/prebaked at temperature 100 °C for 30 min then annealed at temperature 500 °C for 1 h and 2K RPM/3K RPM/4K RPM: thickness variation with the spin rate 2000 RPM, 3000 RPM and 4000 RPM respectively.

## Results and discussion

**Figure 1:**
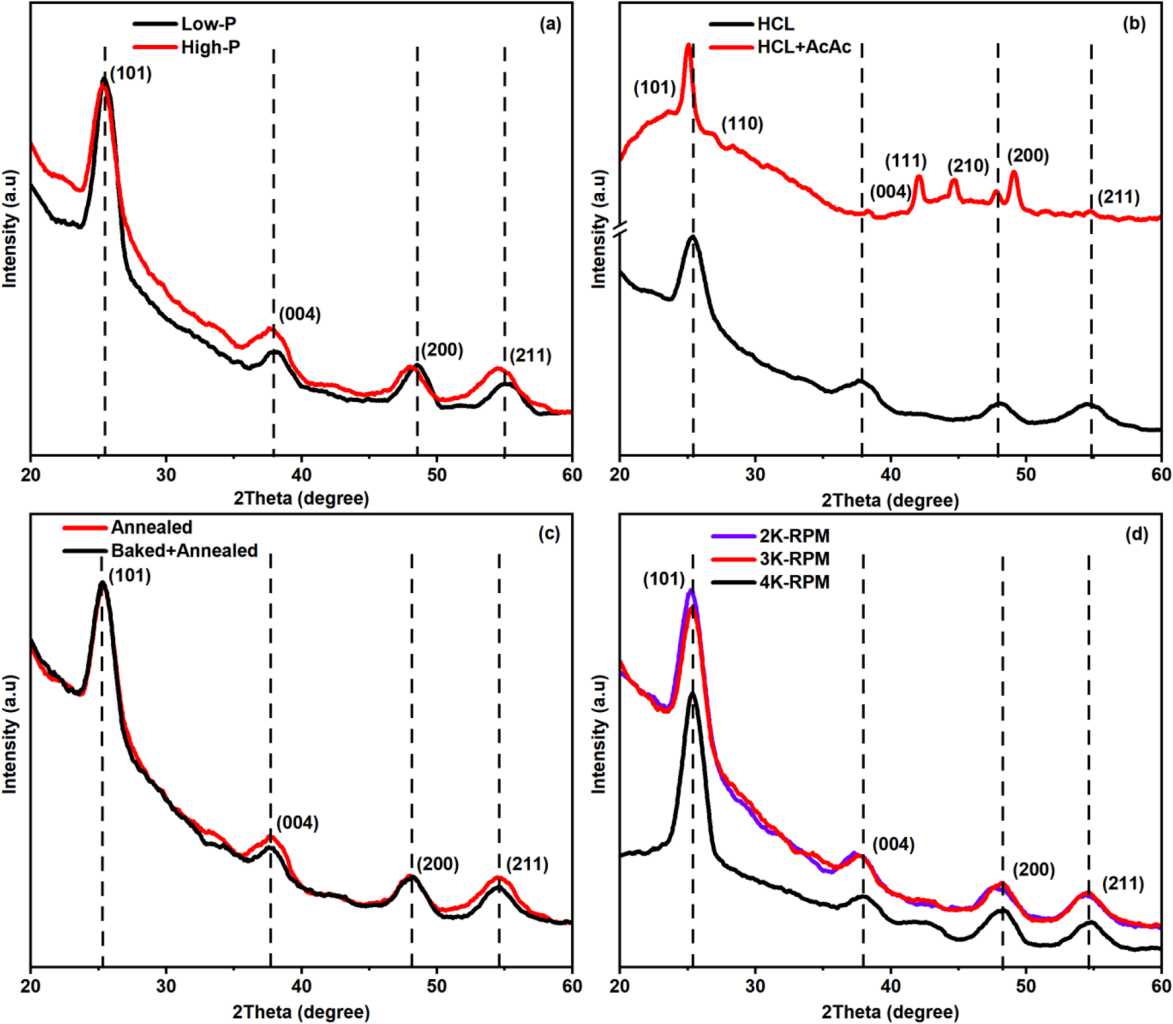
XRD pattern of various TiO_2_/SiO_2_ reflectors prepared by varying precursor concentration (**a**), catalyst concentration (**b**), heat treatment process (**c**) and spin rate (**d**).

By adopting the sol–gel and spin coating processes, the multilayer structures of TiO_2_/SiO_2_ were fabricated and investigated. The prepared dielectric reflectors were treated thermally after the subsequent deposition of each TiO_2_ and SiO_2_ films. The XRD study of various TiO_2_/SiO_2_ multilayer structures was carried out to investigate the crystal structure and phase composition, as depicted in Fig. 1.

Figure 1a–c reveals the XRD patterns of multilayer structures based on 2.5 stacks of TiO_2_/SiO_2_ films i.e. (TiO_2_/SiO_2_)_2.5_ by varying the precursor concentration, catalyst concentration, heat treatment process, and 3.5 stacks i.e. (TiO_2_/SiO_2_)_3.5_ by varying the spin rate. We can observe the XRD patterns of all the samples which are dominated with the single phase of anatase-TiO_2_ except the sample based on ‘HCL+AcAc’ which demonstrates the rutile phase as well. We can also notice that the crystallinity of the TiO_2_ is undisturbed even though the samples were annealed after the successive deposition of each film. The slight broadening of the dominant peak corresponding to the plane (101) can be understood as the presence of the SiO_2_ amorphous phase as well. The Bragg diffraction peaks originated at 2θ = 25.2°, 37.8°, 48° and 55° were ascribed to the planes (101), (004), (200), and (211), respectively. These results coincide with the JCPDS File#21-1272 (anatase-TiO_2_) and the reported works^19–21^. Referring to Fig. 1a, one can observe the a slight broader diffraction peak for the case of ‘Low-P’ sample as compared with the ‘High-P’ one, which indicates the decreased grain size. However, the appearance of the sharp Bragg peak indicating the increased grain size for the sample ‘HCL + AcAc’ based on high acid concentration against the sample ‘HCL’ as shown in Fig. 1b. In addition to the anatase-TiO_2_ phase, we have noticed the appearance of rutile-TiO_2_ phase as confirmed with the peaks originated at 2θ = 27.4°, 41.2°, and 44.4° corresponding to the planes (110), (111), and (210), respectively. These peaks are well matched with the JCPDS File#21-1276. Further, the slight shift of prominent peaks can be noticed which corresponds to the change in the grain size due to the use of high-acidic solution. While the other samples were prepared by using low-acidic solution (i.e. only AcAc) therefore, rutile peaks were absent. The heat treatment process plays a crucial role in the sintering of thin films, and accordingly, various properties such as structural, morphological and optical get affected^22^. The baking of the films before the annealing is helpful to remove the solvent residues and hence, improves the quality of the film. As shown in Fig. 1c, a slight change in XRD patterns of (TiO_2_/SiO_2_)_2.5_ samples can be observed after direct annealing of each TiO_2_ and SiO_2_ films at temperature 500 °C for 1 h as compared to the annealing followed by pre-baking of each TiO_2_ and SiO_2_ films at temperature 100 °C for 30 min. In addition to the heat treatment process, the spin rate is another crucial parameter that controls the thickness of the films and hence, their structural and optical properties. Therefore, we have prepared the TiO_2_/SiO_2_ multilayer structures by varying the spin rate 2000 RPM, 3000 RPM and 4000 RPM to study the optical reflection as discussed later. Figure 1d shows the XRD patterns of the (TiO_2_/SiO_2_)_3.5_ samples prepared at the spin rate 2000 RPM, 3000 RPM and 4000 RPM. These dielectric reflectors exhibited the crystallinity anatase-TiO_2;_ however, the slight deviation in the XRD pattern is noticeable for the samples prepared at the spin rate 2000 RPM, 3000 RPM, and 4000 RPM.

**Figure 2:**
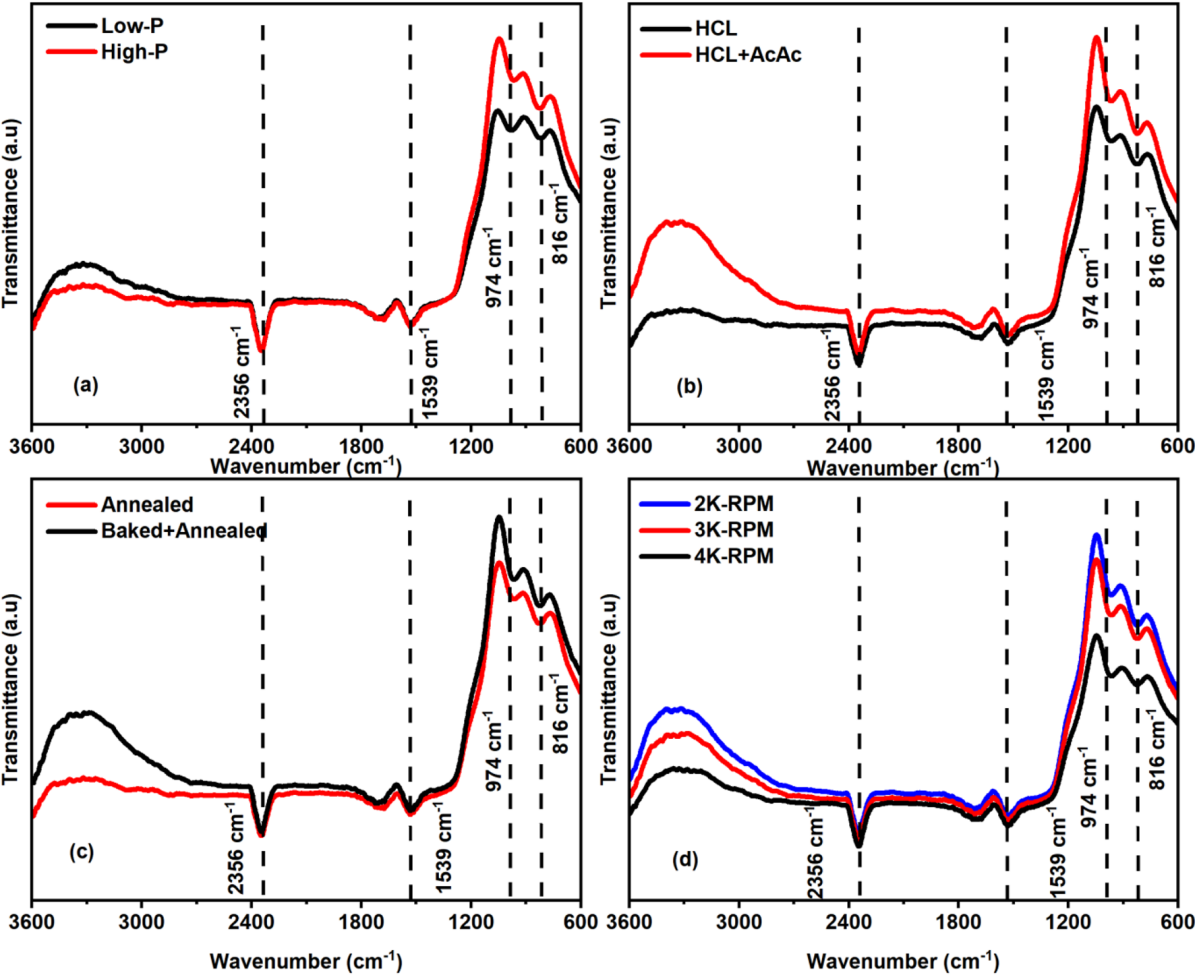
FTIR spectra of various TiO_2_/SiO_2_ reflectors prepared by varying precursor concentration (**a**), catalyst concentration (**b**), heat treatment process (**c**) and spin rate (**d**).

We have explored the investigation to study the chemical compositions present in the samples fabricated by varying the various parameters. Figure 2 depicts the FTIR spectra plotted in the range from 600–3600 cm^−1^ wavenumber. All the samples of TiO_2_/SiO_2_ reflectors reveal the presence of various functional groups prepared by varying precursor concentration, catalyst concentration, heat treatment process, and the spin rate as shown in Fig. 2a–d. The FTIR peak located at 816 cm^−1^ endorses the Si–O–Si asymmetric stretching, whereas the vibration mode of Si–O–Ti can also be noticed at 975 cm^−1^, which corresponds to the overlapping of Si–OH and Si–O–Ti bonds.

Another peak located at 1539 cm^−1^ is attributed to O–H group associated with the bending vibration, whereas the peak originated at 2356 cm^−1^ represents the stretching vibration of O–H-bond. The vibration peaks observed in all the samples were found similar to the reported works^17,23,24^.

**Figure 3:**
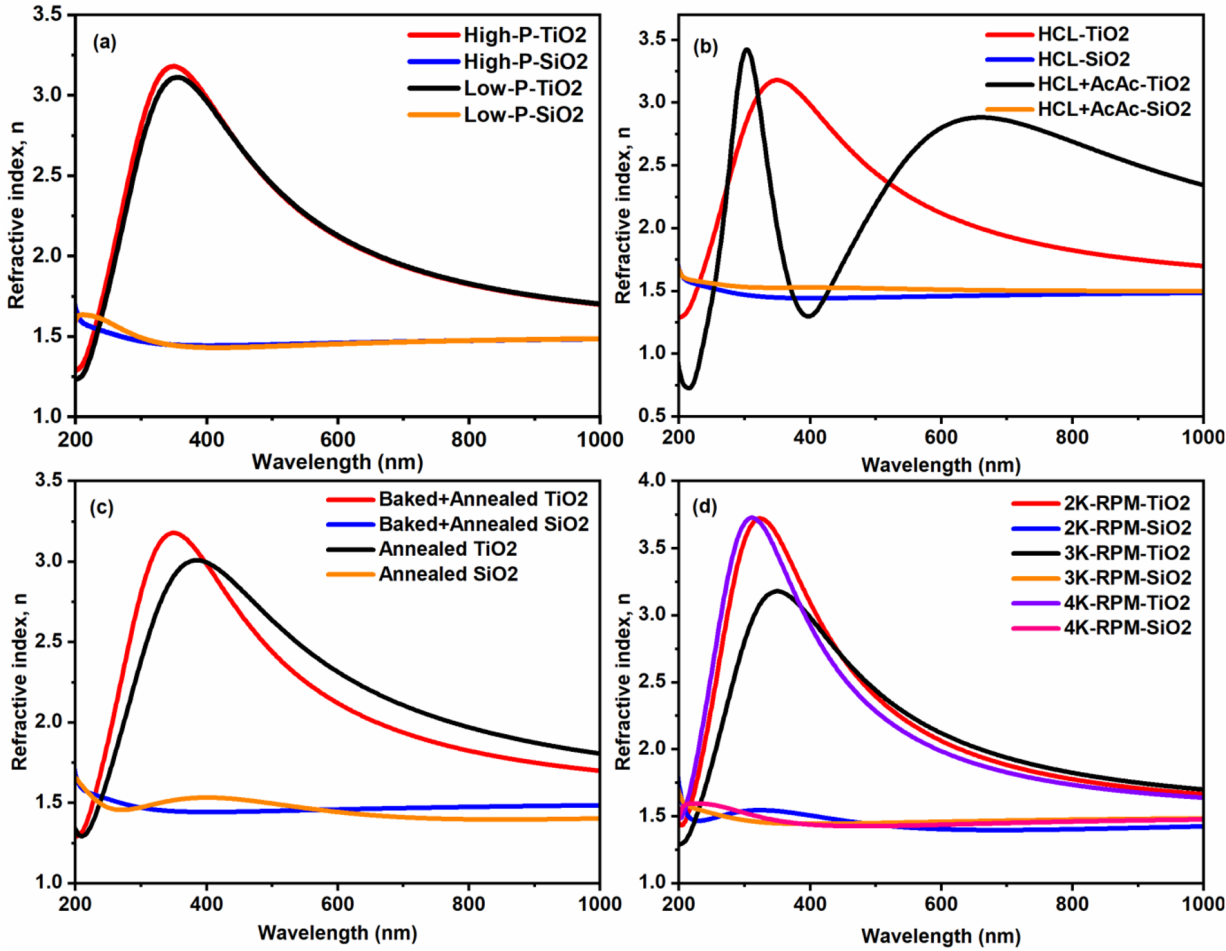
Refractive index versus wavelength graphs of TiO_2_ and SiO_2_ films prepared by varying precursor concentration (**a**), catalyst concentration (**b**), heat treatment process (**c**) and spin rate (**d**).

We have performed the spectroscopic ellipsometry measurement of TiO_2_ and SiO_2_ films. Figure 3 depicts the variation of refractive index values of TiO_2_ and SiO_2_ films prepared by varying the various parameters. Usually, the refractive index is a wavelength-dependent parameter that decreases with an increase of wavelength. The obtained refractive indices were 2.2 and 1.4, corresponding to TiO_2_ and SiO_2_ films at wavelength 766 nm as depicted in Fig. 3a. Besides, the values of refractive indices of TiO_2_ and SiO_2_ films were found in the range from 1.9–2.6 and 1.4–1.5 respectively by varying the precursor concentration, catalyst concentration, heat treatment process, and the spin rate. Remarkably, an increased catalyst concentration (HCL + AcAc for the case of TiO_2_ film) has resulted in the higher refractive index value ‘2.6’. Similarly, Simionescu et al. obtained the higher refractive index value ‘2.7’ of the sputtered TiO_2_ film, and assigned to the presence of mixed anatase and rutile phases^25^. Accordingly, we can also observe the appearance of rutile peaks at 2θ = 27.44°, 41.22°, and 44° corresponding to the planes (110), (111), and (210) in addition to the anatase peaks as shown in Fig. 1b. This indicates that the refractive index of the film also governs by the crystallinity or the presence of crystalline phases in the film.

Furthermore, we can also notice the increased crystallinity of the film with the mixed phases of the anatase and rutile as compared to a film prepared by using ‘HCL’. In general, the value of the refractive index varies from the range of 2.4–2.7 in accordance with the anatase and rutile-TiO_2_ films^26,27^. It is instead to say that the refractive index is influenced by the composition of the film, for example, the amorphous film has lower refractive index value as compared to a crystalline film which possesses higher value^28^. In the same fashion, Danish et al. reported the increased refractive index value from 2.7 to 3.2 due to the improved crystallite size^29^. Likewise, we can observe the improved crystallinity of the film, which resembles the increased crystallite size, as shown in Fig. 1b for the case of HCL + AcAc sample. Similarly, Tanemura et al. obtained the increased refractive index value from 2.8 to 3.9 of the TiO_2_ films fabricated by magnetron sputtering^30^. Our finding is coinciding with these reported works.

**Figure 4:**
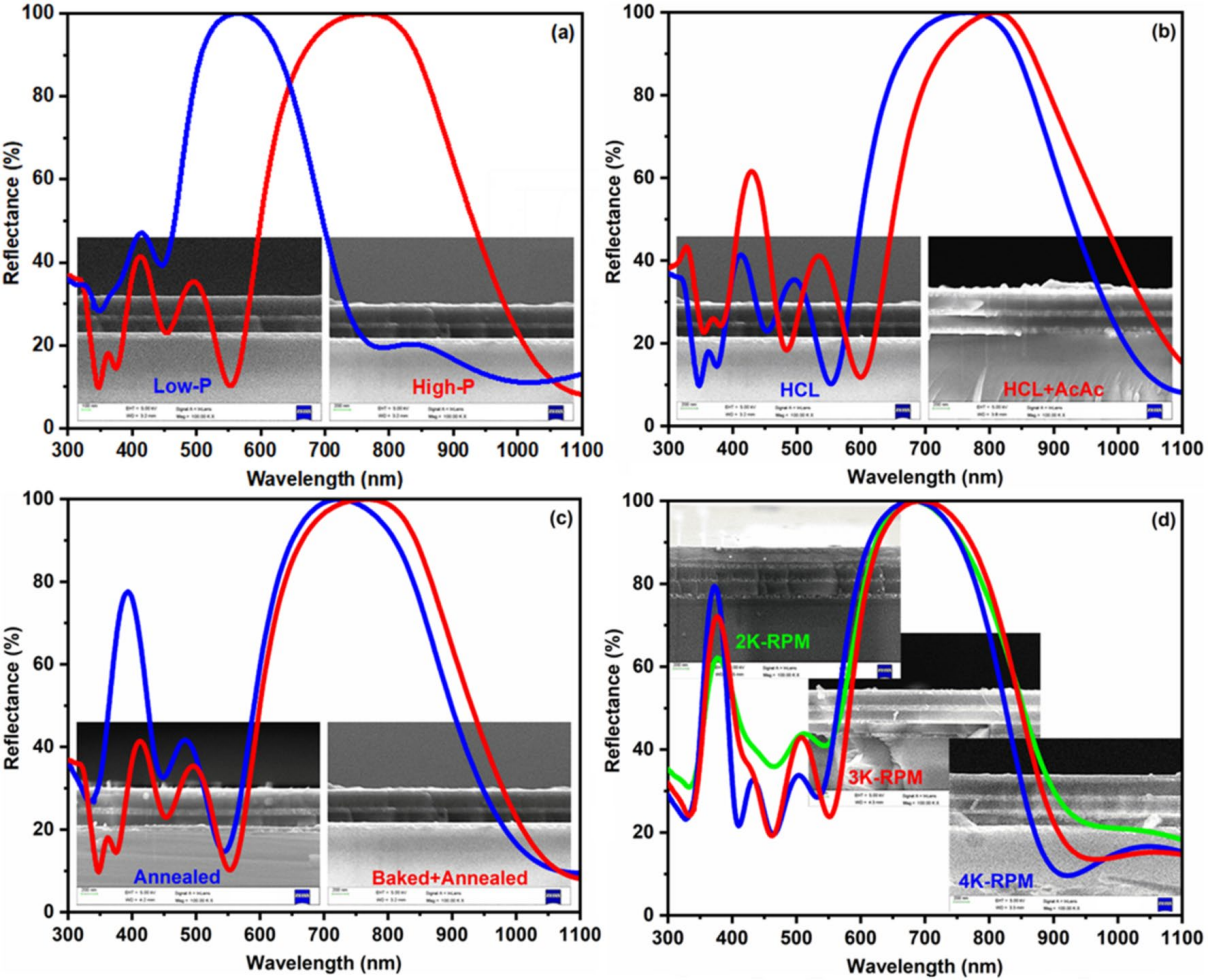
Cross-section FESEM images of TiO_2_/SiO_2_ reflectors prepared by varying precursor concentration (**a**), catalyst concentration (**b**), heat treatment process (**c**) and spin rate (**d**).

Finally, various reflectors were studied for their morphological and optical properties. Figure 4 depicts the cross-section FESEM images and reflectance of TiO_2_/SiO_2_ reflectors. In FESEM images shown in Fig. 4a–d, we can observe the fabrication of periodic layers of TiO_2_ and SiO_2_ films. The brighter and darker layers seen in FESEM images are attributed to the TiO_2_ and SiO_2_ films, respectively. The variation in the thickness can also be noticed as a function of precursor concentration, catalyst concentration, heat treatment process, and the spin rate. The precursor concentration is dominating the reflectance band by shifting it from the center wavelength 567–766 nm in accordance with the low and high precursor concentrations, as shown in the reflectance spectra in Fig. 4a. The increased precursor concentration is not only resulted in the shifting of the reflectance band from visible to near-infrared (Vis–NIR) region but also broaden the reflectance band. An increased precursor concentration yielded the increased grain size, which further resulted in the enhancement of reflectance^31^. Inversely, the use of HCL and the mixed HCL + AcAc catalysts endorse the shifting of the reflectance band from the visible-NIR range. The high acidic solution yielded the shifting of the reflectance band from the center wavelength 766–811 nm as depicted in Fig. 4b. The baking of the individual films prior to annealing reveals the considerable influence on the morphology of the films and the reflectance, as depicted in Fig. 4c. One can see the slight broadening of the reflectance window with its center wavelength 766 nm as compared to 726 nm of the sample annealed directly. Similarly, FESEM images depicted in Fig. 4c shows a fine layered structure after prebaking the samples prior to annealing. The thickness of the alternate films plays a vital role in the shifting of the reflectance band. Figure 4d depicts the effect of spin rate i.e. 2000 RPM, 3000 RPM and 4000 RPM preferred during the coating of the individual films. Depending on the high/low spin rate, the thin/thick film can be fabricated. Accordingly, it results in the shifting of the reflection band towards the shorter/higher wavelength region, as noticed here. Such reflectors also called Bragg reflectors or one-dimensional photonic crystals whose stop band or reflection band shifts to the lower wavelength when the lattice constant i.e. sum of thicknesses of high and low dielectric constants is increased^32^. A similar shifting of the reflectance band can be observed in Fig. 4d with a decreased spin rate. However, the reflector prepared with spin rate 4000 RPM shows the slight shifting of the reflectance curve towards the shorter wavelength, which could be associated with the damaging of an initial layer as visible in Fig. 4d. The center wavelengths of the (TiO_2_/SiO_2_)_3.5_ structures were found to be 680, 696, and 679 nm corresponding to the samples prepared with spin rate 2000 RPM, 3000 RPM, and 4000 RPM. Comparatively, the variation of spin rate/speed directly yields UV–NIR reflection bands. With an increase of spin-coating speed, the NIR reflection band shifts to lower wavelength region along with the enhancement of UV reflection band is noticed.

**Figure 5:**
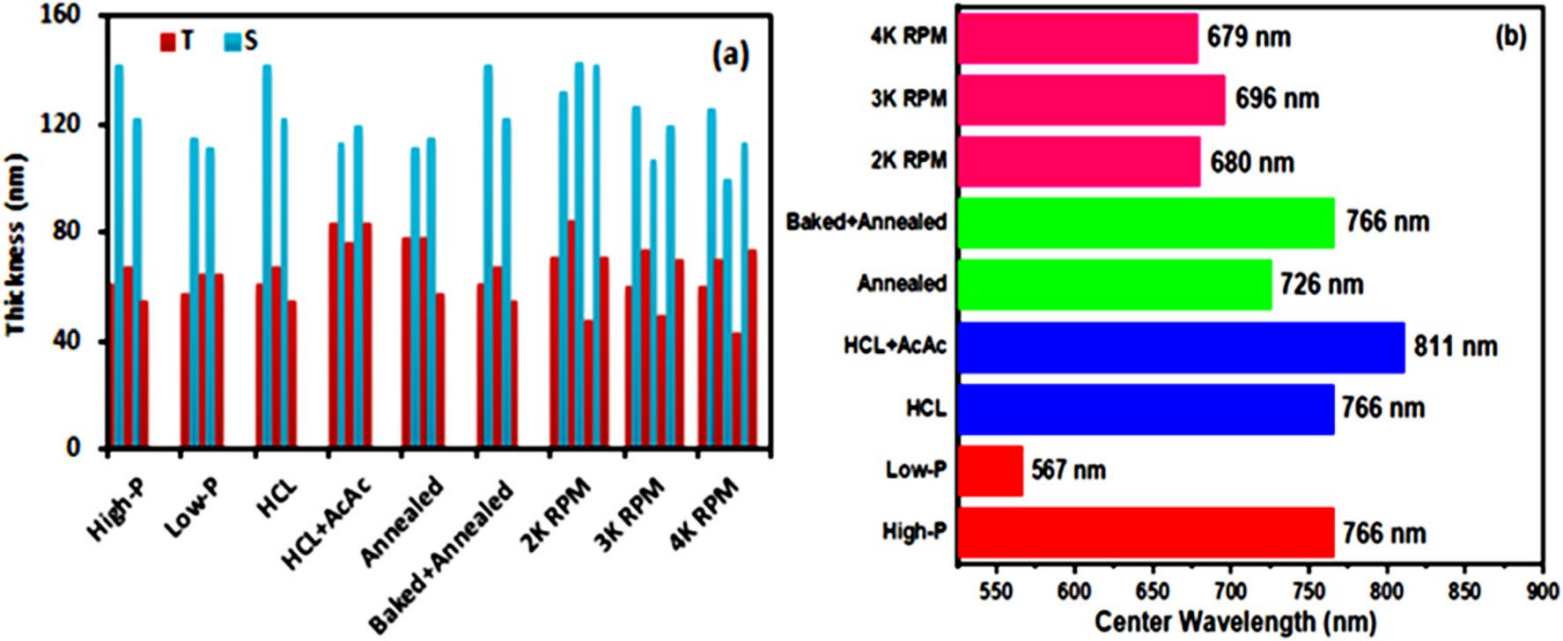
Thickness profile (**a**) and center wavelength variation (**b**) of TiO_2_/SiO_2_ reflectors.

To summarize the various studied parameters in this work, we have plotted Fig. 5. The obtained thickness profile of the various samples of (TiO_2_/SiO_2_)_2.5_ and (TiO_2_/SiO_2_)_3.5_ reflectors prepared by varying the process parameters are summarized in Fig. 5a. The periodic thickness variation of the TiO_2_ (thin) and SiO_2_ (thick) films can be observed for all the cases. As compared to ‘Low-P’ based sample, the ‘High-P’ one shows the increased thickness of the TiO_2_ and SiO_2_ films. Similarly, thickness variations were observed by changing the catalyst concentration and the heat-treatment process. For the case of spin rate investigation, the stacks of the samples were varied to 3.5. The thickness increment of the discrete TiO_2_ and SiO_2_ films is noticeable with the decreased spin rate.

Figure 5b shows the shifting of center wavelength as a function of various process parameters. At most 811 nm center wavelength was noticed for the (TiO_2_/SiO_2_)_2.5_ reflector prepared with the solutions based on mixed acids i.e. HCL + AcAc. Another hand, the least center wavelength 567 nm was obtained for the (TiO_2_/SiO_2_)_2.5_ reflector prepared with the low-concentration based solutions of TiO_2_ and SiO_2_.

**Figure 6:**
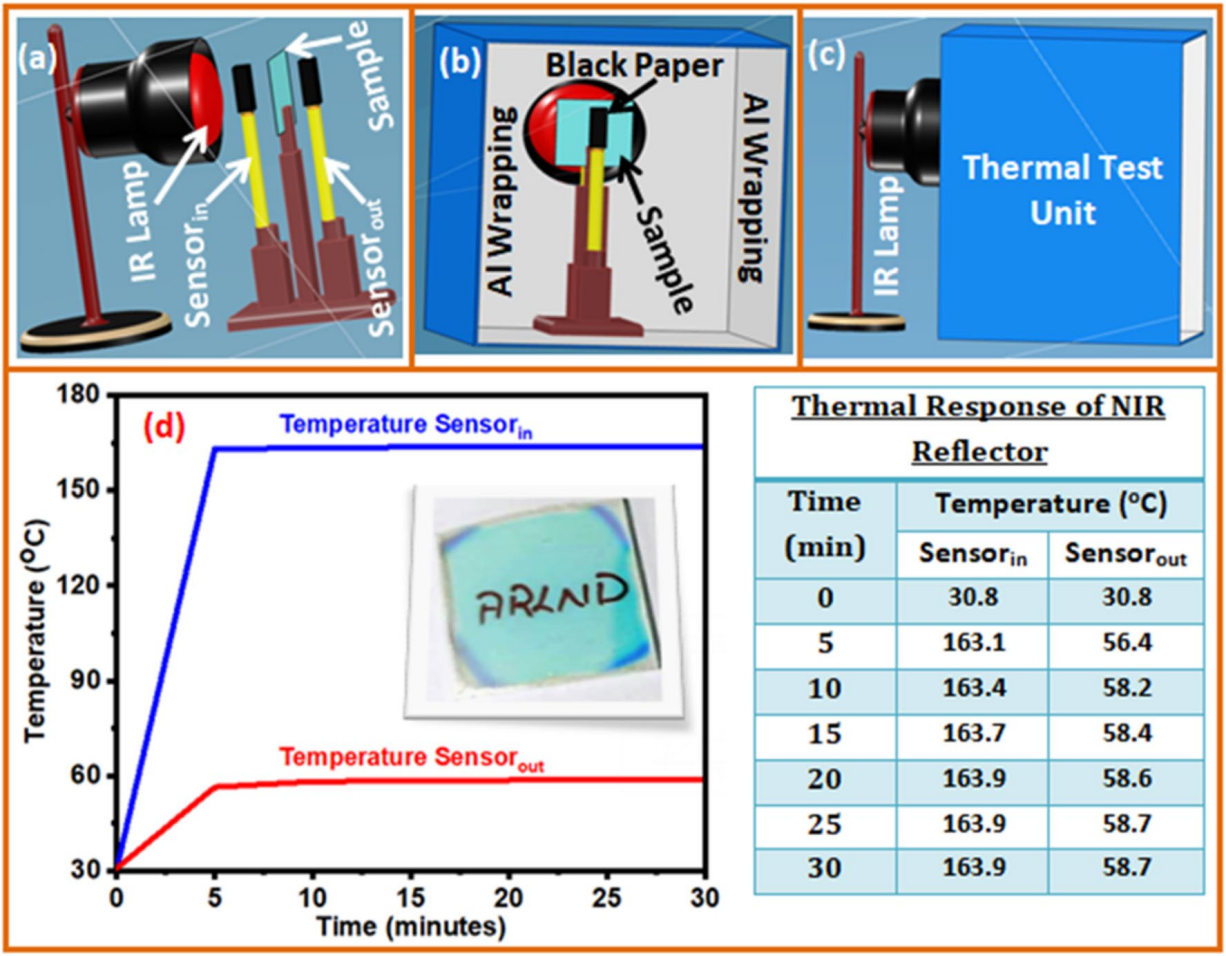
Developed thermal test unit (**a**–**c**) and temperature versus time response of TiO_2_/SiO_2_ reflectors (**d**).

To study the thermal response of the reflector, we have chosen the reflector based on 2.5 stacks of TiO_2_/SiO_2_ layers. The choice of High-P based sample was due to its broadband reflectance in the NIR region having its center wavelength at 766 nm. Figure 6 depicts the developed setup of thermal testing. As depicted in Fig. 6a, it consists of an IR lamp, two temperature sensors (digital thermometers), two pieces of black paper, and a cabinet in which the entire components were assembled. The sample under the test was placed in-between two temperature sensors however, the sensor_in_ was aligned just before the sample to read the ‘in’ temperature while sensor_out_ was placed back to the sample to read the ‘out’ temperature from the sample as shown in Fig. 6b. Both the temperature sensors were wrapped with a piece of black paper to enhance the sensitivity of the sensors. These components and the sample were assembled in a cabinet as shown in Fig. 6c. Figure 6d depicts the temperature versus time response of (TiO_2_/SiO_2_)_2.5_ reflector performed for 30 min. The sensor_in_ and sensor_out_ temperature were noted for every 5 min, as shown right to Fig. 6d. The digital image shown in the inset of Fig. 6d evidenced the transparent film of Peacock blue color. After 5 min, the sensor_in_ temperature was increased to 163.1 °C, while 56.4 °C was noticed by sensor_out_. After 30 min, the temperatures of sensor_in_ and sensor_out_ were found to be 163.9 °C and 58.7 °C, respectively. Pavlichenko et al. prepared and studied the thermal and atmospheric responsive reflectors based on TiO_2_/SiO_2_ films using physical vapor deposition and spin coating methods. They compared the properties of 8.5 bilayers of TiO_2_/SiO_2_ and 4 bilayers structures fabricated by physical vapor deposition and spin coating processes. The optical characteristic of the two reflectors was almost identical. In this view, the sol–gel spin coating suggested as the suitable choice for the fabrication of dielectric reflector as compared to physical vapor fabrication^33^.

Nakamura et al. also studied a bio-inspired multilayer reflector and proposed its self-cleaning and thermal shielding applications. The reflector was transparent with high transmittance in the IR region from 700 to 1000 nm^34^. In the same way, Butt et al. reported the preparation of the dielectric/metal/dielectric reflector and proposed its applications in automobiles and buildings. The prepared reflector suggested for the thermal shielding application, which was transparent for visible light and forbidding IR radiation. Although the prepared reflector had both capabilities, the choice of fabrication using the magnetron sputtering approach was expensive^35^.

By comparing the above literature, our optimized sol–gel spin coating approach is better, which not only reduces manufacturing costs but also minimizes processing time due to the need of fewer coatings. Remarkably, our dielectric reflector evidenced about 105 °C temperature difference between ‘in’ and ‘out’ temperatures by the sensors. The reflector composed of 2.5 stacks endorsed the extraordinary performance, and found prominent for the thermal shielding application in industries. Furthermore, such reflectors are useful for smart window applications by forbidding IR radiation in the summer and preserving room warmth in the winter by prohibiting the heat produced by the room heater.

## Conclusions

We have fabricated TiO_2_/SiO_2_ dielectric reflectors by varying the precursor concentration, catalyst concentration, heat treatment cycle, and spin rate to study their structural, morphological and optical properties. The XRD analysis endorsed the presence of the anatase-TiO_2_ phase in all the samples while the presence of rutile phase also observed in the sample based on high catalyst concentration. The FTIR investigation showed the vibration peaks relating to the TiO_2_ and SiO_2_ films. The refractive index values were noticed in the range from 1.9 to 2.6 and 1.4 to 1.5, corresponding to TiO_2_ and SiO_2_ films. The FESEM analysis evidenced the alternate brighter and darker layers of TiO_2_ and SiO_2_ films, respectively. With the increased precursor concentrations, the reflection bands were found to be wider along with the shifting of their center wavelengths from 567 to 766 nm corresponding to the visible and NIR wavelength regions. A similar shift of the reflection band was observed with the increased concentration of the catalyst with its center wavelength of 811 nm, while 766 nm was achieved after the films were annealed and pre-baked. The spin rate parameter proved to be the dominant factor in shifting the reflection band from a shorter to a longer wavelength region. With this presented analysis, we could achieve 100% reflectance for dielectric reflectors consisting of just 2.5 stacks of TiO_2_/SiO_2_ films. Finally, we have performed the thermal response of the reflector and noticed the extraordinary performance, which suggests its thermal shielding application. In summary, this comprehensive process optimization analysis is useful to tune the optical performance of TiO_2_/SiO_2_ reflectors by adjusting the various parameters for their several applications.
